# Whole course of treatment of autoimmune progesterone dermatitis that had spontaneously resolved during pregnancy: A case report and review of the literature

**DOI:** 10.3389/fimmu.2022.939083

**Published:** 2022-09-07

**Authors:** Yepei Huang, Sha Ye, Xiaoyan Bao, Ru Yang, Jian Huang

**Affiliations:** ^1^Fourth Clinical Medical College of Zhejiang Chinese Medical University, Hangzhou, China; ^2^The Affiliated Hangzhou Hospital of Nanjing Medical University, Hangzhou, China; ^3^Hangzhou Women’s Hospital, Hangzhou, China

**Keywords:** progesterone hypersensitivity, cyclic urticaria, menstrual cycle, progesterone, pregnancy, etonogestrel implant

## Abstract

Anaphylaxis due to autoimmune progesterone dermatitis is a rare but severe allergic disease in women. The clinical manifestations of APD are diverse, and a proper understanding of the disease can help even diagnose and treat it. A case of *autoimmune progesterone dermatitis* related in our department is reported as follows. She developed a rash with severe pruritus that was highly consistent with her menstrual cycle without any trigger 10 years ago. Laboratory tests were unremarkable. But all the symptoms disappeared during her pregnancy and resurfaced after the miscarriage. Two years ago, after a positive progesterone intradermal test confirmed the diagnosis of *PH*, she was given mifepristone, contraceptives(OCPs), and skin embedding treatment, and her symptoms improved.

## Introduction

Autoimmune progesterone dermatitis (APD) is a rare cyclic premenstrual reaction to progesterone produced during the luteal phase of a woman’s menstrual cycle with a variety of presentations including erythema multiforme, eczema, urticaria, angioedema, and progesterone-induced anaphylaxis (1–17). The low prevalence, the high rate of misdiagnosis and the lack of fully effective treatment, although the mortality rate is not high, seriously affects the patient’s daily standard of living. Having found auto-antibodies to progesterone and 17a-hydroxyprogesterone in the serum (18), a condition that may occur during pregnancy or may be aggravated by pregnancy (19), suggesting that the condition may even affect fertility (20). Here we present a rare case of refractory autoimmune progesterone dermatitis with spontaneous resolution of symptoms in pregnancy. Also this is the first report of experience with effective treatment of progesterone-induced dermatitis with skin embedding treatment.

## Case presentation

A 35 - year - old unmarried female was transferred to the department of gynecology clinic of our hospital. The patient had a gynecologic history of menarche at the age of 15 and reported 30-day menstrual cycles. The patient reported periodic rash and pruritus for ten years and asked for medical history. A long-term practice in the medical aesthetic industry, the patient reported no obvious etiology before the onset of the disease, no other basic medical history, and no history of exogenous hormone use before the onset of the disease.

**Figure 1:**
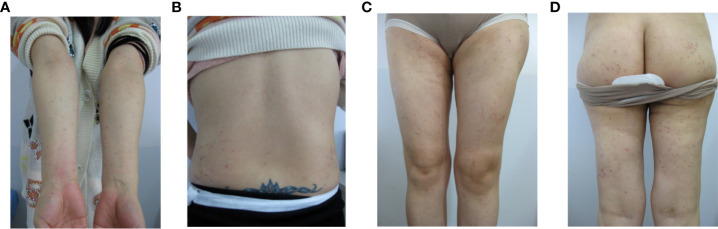
The polymorphic cutaneous manifestations of progestogen hypersensitivity. (**A**: arms, **B**: back, **C**: legs, **D**: hips).

During this period, the patient had a pregnancy, the symptoms disappeared during pregnancy, and relapsed after abortion. The characteristics were the same as before. Physical examination showed that the rash with lesions consisting of scattered erythema, papules, herpes colliculus, scratch, scab and a large number of secondary pigmentation spots mainly localized on the limbs and buttocks (**Figures 1A–D**). The flares started on days 7 of the menstrual cycle and resolved on days 3 of menstruation.

In the past 10 years, she repeatedly visited the dermatology department with eczema and was treated with multiple medications including antihistamines and dexamethasone, but there was no significant improvement. Until a gynecologist suspected APD and performed intradermal tests with medroxyprogesterone up to 50 mg/mL in aqueous solution. The results were positive.

**Figure 2:**
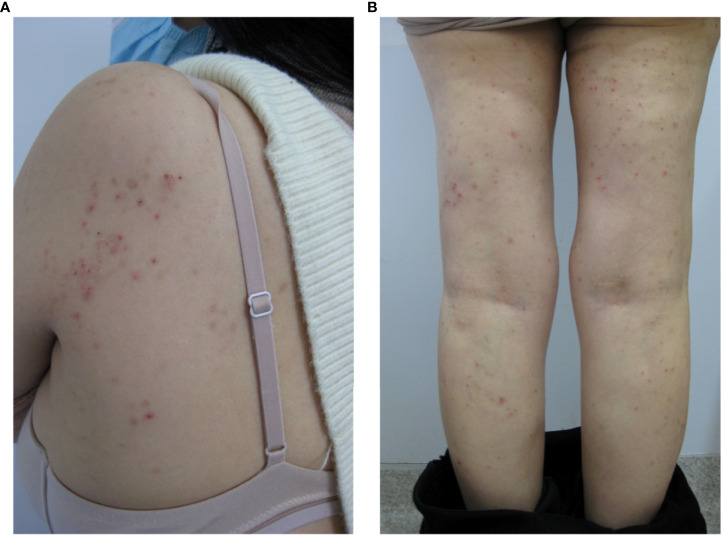
Manifestations of shoulder **(A)** and leg **(B)** lesions after 3 months of skin embedding treatment.

Following the diagnosis, the patient received a mifepristone tablets (25mg) therapy for two months and discontinued all remaining medications. The first cycle was very effective, with more than 90% reduction in rash area and no significant pruritus, but the second cycle had a recurrence of symptoms to the same extent as before the drug was taken. Because the effect is not good, she changed to take combined oral contraceptives therapy with drospirenone (3 mg) and ethinyl estradiol (0.03 mg) for a total of 5 cycles. However, the symptoms fluctuated. Considering the patient’s need for contraception and occasional missed doses, the follow-up doctor suggested relying on Etonogestrel Implants with Etonogestrel(68mg) skin embedding treatment. After 3 menstrual cycles, the hip and thigh symptoms were significantly improved with 1/3 reduction in seizure area and poor improvement in both arms (**Figures 2A, B**). The patient’s next steps are still being discussed.

## Discussion

Autoimmune progesterone dermatitis (APD) was first discovered in 1964 (21), which is a rare cyclic premenstrual reaction to progesterone occurs exclusively in women of childbearing age. Synthesizing the published literature now (**Tables 1**, **2**), the eruption of autoimmune progesterone dermatitis varies morphologically. The most typical clinical manifestation of the disease is a rash that regularly worsens or remits with the menstrual cycle. The rash can manifest as urticaria, angioedema, erythema multiforme, eczema, fixed drug rash, etc. (1–17). The lesions are characteristically symmetrical and occur on the face, trunk, and extremities. Patients often feel itchy, and some have systemic symptoms, such as chest tightness, shortness of breath and anaphylactic reaction. Stomatitis and mucosal lesions are less common. On physical examination, ruptured blisters leading to crusting and hyperplastic hypopigmented scarring are also seen. The lesions usually occur 3 to 10 days before menstruation, resolve spontaneously after menstruation. However, the onset of symptoms triggered by exposure to exogenous progesterones is not necessarily related to the menstrual cycle. Some women with APD will have irregular menstrual periods. Available studies suggest that APD may affect reproductive function (20).

**Table 1:** Characteristics of patients with APD reported in English literature from 2013 to 2022 (N=49).

Value	
*Medical history*		
(1) Age of onset (y), mean (range)		30 (12-50)
(2) Endogenous progesterone trigger, %		66
Exogenous progesterone trigger, %		34
		12 OCP
		10 After delivery
		4 Use progesterone during pregnancy
		4 IVF
		2 HIV
		2 IUD
(3) Relation to menses,%	88% coincide with the menstrual cycle	
(4) Organ involvement,%		
Cutaneous		92 Dermatitis
		20 Angioedema
Pulmonary		20
Gastrointestinal		12
Cardiovascular and neurologic		12
Abortion		4
*Diagnostic modality*	Number of Use cases	Positive rate,%
Skin prick test	33	91
Intradermal	1	100
Progesterone patch	1	100
sIgE	1	100
ND	13	

IUD, Intrauterine device; ND, no data.

**Table 2:** Treatment modalities and outcomes of patients with APD reported in English literature from 2013 to 2022 (N=49).

Treatment	Ourcome (N)		
	cured	attenuated	failed
OCP	8	5	2
GnRH agonist	4	4	0
17-α-alkylated steroid	0	7	0
Antihistamine	3	1	1
Tamoxifen	1	0	0
Bilateral salpingooophorectomy	5	0	0
Glucocorticoid	0	1	0
Desensitization	2	0	1
Medroxyprogesterone	1	1	0
Omalizumab	1	0	0
Stop using exogenous progesterone	3	0	1

GnRH agonists, gonadotropin-releasing hormone agonists.

### Pathogenesis of APD

Sex hormones are closely related to the development and regulation of the immune system. Both the innate and adaptive immune systems have sex hormone receptors and respond to hormonal signals, and dysregulation of these mechanisms can lead to immune-mediated diseases (22–26). Estrogen receptors are widely dispersed in many immunomodulatory cells and have a role in regulating immune cell function. Estrogen has an inhibitory effect on Th1 cells, induces activation and proliferation of Th2 cells, and therefore facilitates the allergic response caused by Th2 polarization and promotes the conversion of immunoglobulin (Ig)-producing B cells, resulting in increased levels of antibodies and inflammation-related cytokines in the body, such as IgG and IgE (27, 28). In addition estrogen promotes the degranulation of basophils, and mast cells known as immediate hypersensitivity effector cells. eNOS expression and NO production are regulated. Thus, estrogen levels not only affect the immune system but also play an important role in allergic diseases. Estrogen allergy has also been associated with premenstrual dermatitis, and has been reported to be due to dendritic cell mediation, and dendritic cells are potent antigen-presenting cells involved in the induction of primary immune responses (29, 30). Androgens are generally considered to have immunosuppressive effects (31). While progesterones apparently have immunomodulatory effects on the immune system, particularly on T cells and T cell subsets (32, 33).

However, the pathogenesis of APD in humans is still not particularly clear. Exogenous progesterone exposure is an important cause of morbidity, such as those used for oral contraception pills(OCPS) (33) or *in-vitro* fertilization (IVF) (1). There is now a theory that exogenous progesterone stimulates the body to form progesterone-specific IgE antibodies (34), which could be identified by enzyme-linked immunosorbent assay (ELISA) (35). Due to the cross-linked activation of mast cells by these antibodies resulting in Autoimmune progesterone dermatitis (34). Evidence of basophil and mast cells (MCs) activation using functional assays also supports an IgE-mediated immune response (34).Progesterone has also been suggested to contribute to pathogenesis through mechanisms such as delayed hypersensitivity through G protein-coupled receptor modulation of TH2 cells, through activation of progesterone membrane receptor α (PHR α) on CD8+ cells, and through immune complex-mediated theory, among others.

The exogenous progesterone hypothesis does not explain all cases. Most of the reported patients had no history of exogenous progesterone administration. The most common mechanism of sensitization nowadays is cross-sensitization to corticosteroids that are structurally similar to progesterone (36). Another mechanism for sensitization may be that some women can only tolerate low levels of the hormone (37). In a normal menstrual cycle, progesterone levels in the luteal phase can be 10-35 times higher than in the follicular phase (38). Not to mention that during pregnancy, progesterone can be even thousands of times higher than the pre-pregnancy state. In certain physiological states, they produce an inflammatory response as levels rise (37), with high levels of progesterone varying from patient to patient, which could explain the cutaneous eruption appears or is exacerbated during the luteal half of the menstrual cycle and during pregnancy and postpartum. Besides, in cultured human keratinocytes, the expression of progesterone receptor (PR) and the detection of PR mRNA transcription may explain the advantages of skin lesions in APD to some extent (39). The diversity of the clinical manifestations of the disease also suggests that the pathogenesis cannot be the only one and needs to be explored.

However, the reported patient’s dermatitis disappeared completely during pregnancy. Bierman et al. suggested that this may be due to a weakened maternal immune response to progesterone during pregnancy (19); Stephen et al. suggested that the rising progesterone during pregnancy caused hormonal desensitization (40). More research is needed into the mechanisms involved.

Autoantibodies to progesterone and 17a-hydroxyprogesterone were found in the serum (18), which may occur during pregnancy or may be exacerbated by pregnancy (19). In addition, in a study (18), 29 women with recurrent miscarriages were screened for estrogen and progestin allergy by intradermal testing; most had positive skin tests and all of the controls were negative. Sixteen of them had successful pregnancies and live births after desensitization (41), with five of them having two successful deliveries. This suggests that indicates that APD may even affect fertility (20) and that recurrent miscarriages may be associated with inappropriate immune-mediated responses to estrogen and progestin.

Immune factors undoubtedly play an important role in many stages of female reproduction:(1)self-tolerance and inflammatory response; and (2) factors that lead to a dysregulated maternal immune response to specific fetal or trophoblast antigens (which may be partly genetically determined) (42). Maternal and fetal immunology is difficult to study in humans, and almost all evidence for the role of the immune system in the maintenance of human pregnancy comes from studies comparing relevant immune biomarkers in normal and pathological pregnancies. There is substantial evidence that human leukocyte antigens (HLA), anti-sperm antibodies, integrins, leukemia inhibitory factor (LIF), cytokines, antiphospholipid antibodies, endometrial adhesion factors, mucin (MUC1), and uterine natural killer cells all contribute to reproductive failure (43). The development of recurrent spontaneous abortion (RSA) is also associated with immune factors (44), and immune-related RSA can be divided into two main categories: autoimmune and alloimmune types (45). The autoimmune type is mainly associated with autoimmune diseases such as antiphospholipid syndrome (APS) and systemic lupus erythematosus (SLE) and associated autoantibodies.The autoantibody most closely associated with recurrent miscarriage is antiphospholipid antibodies (APA). The antibody APA may exacerbate thrombosis at the placental interface, leading to embryonic ischemic death and miscarriage (43, 44, 46). APA may also inhibit trophoblast function, affecting the embryonic implantation process, as well as preventing the transformation of cytotrophoblasts into syncytial trophoblasts, leaving the fetus with an inadequate supply of nutrients. The alloimmune phenotype is mainly related to the balance of immune tolerance in pregnancy and includes two categories of protective antibody defects and lymphocyte disorders. Most patients with RSA have abnormal NK activity,which may be due to the interaction of killer immunoglobulin-like receptors (KIR) on uterine natural killer (uNK) cells with HLA-C antigens expressed by trophoblast cells (47), thereby suppressing trophoblast cells, which is an important cause of recurrent miscarriage. In addition, CD8+ suppressor T cells, on the other hand, may promote fetal immune tolerance by suppressing the maternal immune system to reject the fetus (48); Th17/Treg cell balance is essential to maintain the homeostasis of the immune system, with Treg cells and their associated cytokines favorable to pregnancy outcome and Th17 cells and their associated cytokines unfavorable (45, 49). Male-specific small histocompatibility (HY) antigens also cause RPL, especially secondary recurrent miscarriage (SRM) (44). Of cause, the present researches are limited with studies being small. How APD affects female reproductive relationships through the immune system needs to be further explored.

### Diagnosis of APD

The diagnosis of APD is currently exclusionary, excluding other steroid syndromes, drug-induced diseases other than progesterone, and various primary dermatologic conditions. The heterogeneity of APD symptoms emphasizes the importance of a systematic and thorough history and physical examination during the patient’s visit. The clinical diagnosis of the disease is currently based on skin lesions that vary with the menstrual cycle and symptoms that are triggered by exposure to exogenous progesterones, combined with a progesterone intradermal test or progesterone challenge or several *in vitro* assays, can determine the diagnosis. The skin prick test with full concentration of progesterone (50 mg/mL) dissolved in oil is commonly used clinically. If the result is negative but there is a high clinical suspicion, an intradermal test may be performed, starting with a dilution of 0.01% concentration and increasing to a maximum of 1% concentration [diluents can be ethanol or oil based (50)], together with appropriate saline negative and positive histamine controls. A positive skin test can be rapid (within 30 minutes) (51, 52) or late (24-96 hours) (1, 53). Dinah et al. did a study where only half of the patients had a positive skin test (51). Ethanol or oil diluents may cause irritation responses and end up with false positive results. Replacing progesterone skin testing with water-soluble progesterone is one of the latest solutions (54). Therefore, a negative test cannot be completely ruled out, and should be combined with clinical manifestations to make a comprehensive judgment. Progesterone Challenge (17) is also available clinically. However, single dose of graded challenge with oral progesterone does not always elicit symptoms and often requires multiple updates to elicit a response, which in some individuals may result in severe and prolonged skin eruptions (50) leading to unnecessary morbidity and should therefore be used with caution in patients with a history of progesterone-induced reactions. Progesterone challenge experiments may not work if the patient has a very clear history of onset after exogenous progesterone exposure (50). Based on current clinical data, progesterone patch is not effective and is not routinely performed. Alternatively, intramuscular injection of progesterone using a concentration of 12.5 to 25 mg of progesterone can be used as an alternative diagnostic test (55). Both intramuscular or oral progesterone can induce the disease. However, it is not carried out much in clinical practice due to pain at the intramuscular injection site and poor patient cooperation (34, 55). *In vitro* tests are also increasingly important for detecting APD, such as the leukocyte histamine test (LHR) to assess histamine release from basophils and the interferon-gamma release test to assess progesterone-related T-cell-mediated activity, as well as the enzyme-linked immunosorbent assay (ELISA) to assess progesterone-specific IgE (sIgE), as mentioned earlier (34, 35, 56, 57). No test is 100% accurate and when we encounter a patient who has a negative test but whose clinical presentation is highly consistent with APD, we can use empirical treatment or desensitization and if the treatment is effective, this is a successful therapeutic diagnosis.

### Therapies for APD

**Figure 3:**
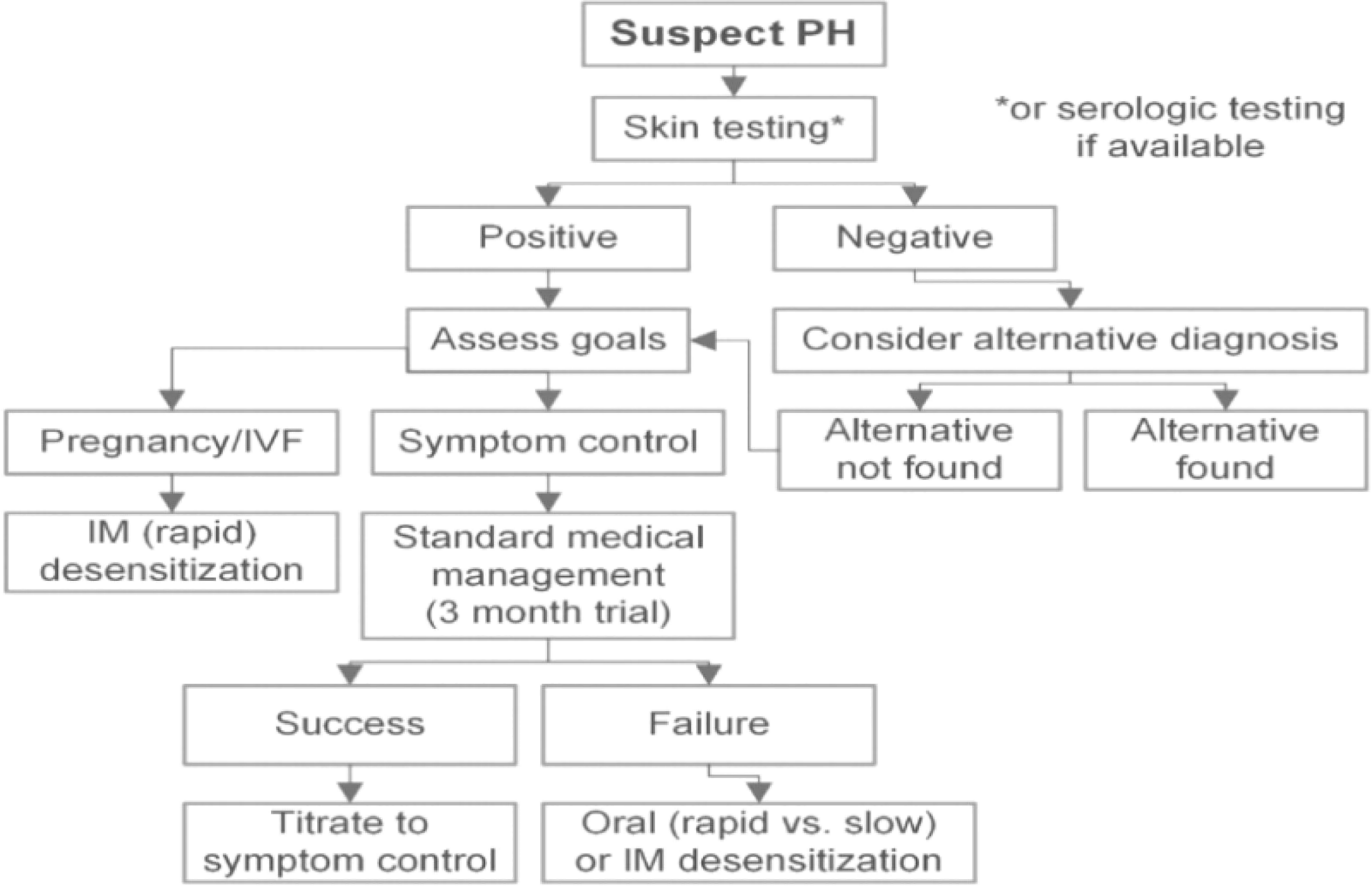
Management (51).

Therapies for APD are currently available and there are various options depending on the patient’s existing condition and her current needs (**Figure 3**)

Many of APD patients are misdiagnosed as dermatologic diseases and are often given antihistamines of the H1 or H2 class (58) and glucocorticoids, which are the recommended starting regimen for APD patients and adjuncts to other treatments, and a small percentage of patients have improved symptoms (18, 59). The most classical principle of medication is the suppression of ovarian ovulation and the reduction of progesterone levels in patients in the luteal phase. The commonly used drug is the contraceptive pill, and many successful treatment cases have been reported; however, some patients cannot tolerate the low levels of progesterone in the pill and need to be treated with other drugs. In addition, dermal embedding treatment can slowly release a small amount of progesterone to inhibit ovulation. This contraceptive contains only progesterone and no estrogen, so the side effects are less than those of oral contraceptives, which are less reported in the literature.

Tamoxifen, a non-steroidal anti-estrogenic drug. Cured cases with this drug have been reported (17, 60, 61). However, it is suitable for use in menopausal women due to a significant increase in side effects and menopause-like symptoms, such as hot flashes and sweating, vaginal dryness, irritability, osteoporosis, and increased incidence of cardiovascular disease due to long-term lack of estrogen (62).

Intramuscular and intranasal gonadotropin-releasing hormone (GnRH) agonists, inhibit ovulation by interfering with pituitary-hypothalamus feedback regulation (63), with high cure rates (64, 65). However, similar to the side effects of tamoxifen, it cannot be used for a long period of time and the course of treatment is usually less than 6 months.17-a-alkylated steroids (66) also depresses the pituitary-hypothalamic response and interferes with gonadal hormone receptors (7). One case was reported in the literature (18) in which APD symptoms were improved while danazol was used to treat endometriosis. This inhibition is thought to be the result of reduced IgG production during cortisol treatment. Stanozolol and danazol (67) have been used successfully in combination with glucocorticoids for premenstrual flares of chronic urticaria. Side effects of the drugs include Hirsutism, mood changes and increased liver function tests, among others. More extreme methods include oophorectomy, which can completely cure the disease (68), but also results in loss of fertility and is generally not considered in women of childbearing age or who still have childbearing requirements.

Of note, almost all patients with autoimmune progesterone dermatitis treated with ovulation-suppressing estrogen preparations showed improvement, and the pharmacological effects of Conjugated equine estrogen and ethinyl estradiol therapy are mediated by interactions between estrogen receptor subtypes. Occasionally, the therapy is discontinued because of poor tolerance or chronic vaginal bleeding, and long-term high levels of estrogen supplementation increase the risk of endometrial cancer (69). Heffler et al. tried the use of omalizumab – an anti-IgE monoclonal antibody approved for severe allergic asthma and severe chronic spontaneous urticaria-and successfully cured one patient (70), which provides a new idea for the treatment of this disease.

Progesterone desensitization is also a good option. Commonly used clinical desensitization protocols include oral, intramuscular (IM), and intravaginal suppository protocols. For patients who need to tolerate high doses of progesterone, such as those attempting IVF, in this case, rapid desensitization therapy can help patients quickly build tolerance to progesterone (14, 51), in preparation for embryo transfer. Both intramuscular (IM) and vaginal protocols have been documented to successfully lead to pregnancy (2, 14, 21). Rapid progesterone desensitization is also used to relieve urticaria and angioedema triggered by progesterone exposure (51). Rapid or slow desensitization regimens can be tried to bring progesterone to stable levels for patients who are not satisfied with standard treatment regimens or who want to improve their quality of life. It is important to note that after successful desensitization, patients must continue to take oral OCP to maintain a stable progesterone status and avoid re-desensitization. In patients with recurrent miscarriage we would attempt to supplement with progesterone, which in the case of sex hormone hypersensitivity may no longer play its physiological role in supporting pregnancy, leading to an outcome of miscarriage (20), whereas desensitization may have a significant positive effect on fetal growth and subsequent pregnancy outcome in such patients.

APD is a rare disease that is often confused with skin diseases due to its various clinical manifestations and delays in treatment, which requires an in-depth understanding and differential diagnosis by gynecologists and dermatologists. In addition, as with other female reproductive hormone allergy syndromes, little is known about this disease, and specific pathological mechanisms, highly accurate tests and more treatment options are waiting to be discovered. In addition to our patient, her reproductive needs remain, and we will continue to try other appropriate treatment modalities.

## Data availability statement

The original contributions presented in the study are included in the article/supplementary material. Further inquiries can be directed to the corresponding author.

## Ethics statement

Written informed consent was obtained from the individual(s) for the publication of any potentially identifiable images or data included in this article.

## Author contributions

YH completed the main body of the manuscript. YH and JH made decisions about the entire treatment process and provided the main ideas for writing the final article. SY, XB, RY, and JH have supplemented and modified parts of the manuscript and participated in the collation of patient data. All authors contributed to the article and approved the submitted version.

## Conflict of interest

The authors declare that the research was conducted in the absence of any commercial or financial relationships that could be construed as a potential conflict of interest.

## Publisher’s note

All claims expressed in this article are solely those of the authors and do not necessarily represent those of their affiliated organizations, or those of the publisher, the editors and the reviewers. Any product that may be evaluated in this article, or claim that may be made by its manufacturer, is not guaranteed or endorsed by the publisher.
